# Inequalities in maternal health care utilization in Benin: a population based cross-sectional study

**DOI:** 10.1186/s12884-018-1846-6

**Published:** 2018-05-31

**Authors:** Sanni Yaya, Olalekan A. Uthman, Agbessi Amouzou, Michael Ekholuenetale, Ghose Bishwajit

**Affiliations:** 1 School of International Development and Global Studies, University of Ottawa, Ottawa, Canada; 2 Warwick Centre for Applied Health Research and Delivery (WCAHRD), Division of Health Sciences, Warwick Medical School, University of Warwick, Coventry, CV4 7AL UK; 3 Bloomberg School of Public Health, Johns Hopkins University, Baltimore, MD USA; 4 Department of Epidemiology and Medical Statistics, Faculty of Public Health, College of Medicine, University of Ibadan, Ibadan, Nigeria

**Keywords:** Antenatal care, Postnatal care, Contraceptive use, Institutional delivery, Inequalities, Benin, Demographic and health survey, Cross-sectional study

## Abstract

**Background:**

Ensuring equitable access to maternal health care including antenatal, delivery, postnatal services and fertility control methods, is one of the most critical challenges for public health sector. There are significant disparities in maternal health care indicators across many geographical locations, maternal, economic, socio-demographic factors in many countries in sub-Sahara Africa. In this study, we comparatively explored the utilization level of maternal health care, and examined disparities in the determinants of major maternal health outcomes.

**Methods:**

This paper used data from two rounds of Benin Demographic and Health Survey (BDHS) to examine the utilization and disparities in factors of maternal health care indicators using logistic regression models. Participants were 17,794 and 16,599 women aged between15–49 years in 2006 and 2012 respectively. Women’s characteristics were reported in percentage, mean and standard deviation.

**Results:**

Mean (±SD) age of the participants was 29.0 (±9.0) in both surveys. The percentage of at least 4 ANC visits was approximately 61% without any change between the two rounds of surveys, facility based delivery was 93.5% in 2012, with 4.9% increase from 2006; postnatal care was currently 18.4% and contraceptive use was estimated below one-fifth. The results of multivariable logistic regression models showed disparities in maternal health care service utilization, including antenatal care, facility-based delivery, postnatal care and contraceptive use across selected maternal factors. The current BHDS showed age, region, religion were significantly associated with maternal health care services. Educated women, those from households of high wealth index and women currently working were more likely to utilize maternal health care services, compared to women with no formal education, from poorest households or not currently employed. Women who watch television (TV) were 1.31 (OR = 1.31; 95% CI = 1.13–1.52), 1.69 (OR = 1.69; 95% CI = 1.20–2.37) and 1.38 (OR = 1.38; 95% CI = 1.16–1.65) times as likely to utilize maternal health care services after adjusting for other covariates.

**Conclusion:**

The findings would guide stakeholders to address inequalities in maternal health care services. More so, health care programmes and policies should be strengthened to enhance accessibility as well as improve the utilization of maternal care services, especially for the disadvantaged, uneducated and those who live in hard-to-reach rural areas in Benin. The Benin government needs to create strategies that cover both the supply and demand side factors at attain the universal health coverage.

## Background

The steps towards achieving the third United Nations (UN) Sustainable Development Goals (SDGs), to reduce maternal morbidity and mortality and achieve universal health coverage to include access to essential health care services by 2030 have been a great issue in developing countries, even with the existence of health care interventions. Though there are numerous health care implementation projects to promote safe motherhood worldwide, maternal morbidity and mortality remain a notable hitch in health care programme and policy making particularly in low-income countries. In spite of the vast efforts by the global community to lessen the burden of mortality as a result of pregnancy and delivery, the rate of death due to pregnancy related complications is worrisome [1]. Developing countries have been reported to account for about 99% of the global maternal mortality, while sub-Saharan Africa (SSA) countries record approximately 62% and having Maternal Mortality Ratio (MMR) of 510 maternal deaths per 100,000 live births [2, 3]. The challenge of unfair distribution of health care services is gaining global attention in the area of public health, with evidence of the disadvantaged sections of the society, having worst health conditions [1]. Like other sub-Saharan countries, Benin is having an unfair share in maternal health care. A country with Total Fertility Rate (TFR) of 5.3, is ranked the 34th in the world with maternal death [4].

Inadequate of access to antenatal, intrapartum and postnatal health care services are among the prominent reasons for high maternal and child morbidities and mortalities in SSA and the world at large [5, 6]. Maternal health care services continue to be important indicators for monitoring the improvement of maternal health outcomes, as well as maternal mortality. In addition, antenatal care, institutional health delivery with skilled birth attendant, and postnatal care strengthen prompt management and treatment of pregnancy related complications to reduce maternal mortality. Besides the benefits of institutional based delivery in the prevention of maternal death, more women give birth utilizing alternative places such as home and Traditional Birth Attendants (TBA) who are not knowledgeable in modern obstetric care [7]. One of the major pillars of the Safe Motherhood Initiative is antenatal care, which helps to provide interventions that are essential for positive pregnancy outcomes [8]. World Health Organization (WHO) remark that receiving antenatal care not less than four times increases the odds of receiving valuable health care promotion and preventive maternal health care interventions during antenatal visits [9, 10]. Furthermore, family planning is also a vital indicator of the Safe Motherhood Initiative to reduce pregnancy related complications and death in developing countries [10].

Essential emergency obstetric health care services are required to access key equitable resources across regions, socio-economic strata and geographical locations [11]. Maternal health care services encompass a wide range of clinical procedures and care provided to women during pregnancy. As a matter of necessity, all pregnant women should have access to quality antenatal care regardless of their economic, cultural, geographical and social background. Interestingly, antenatal care performs a crucial role in ensuring a healthy baby and mother during pregnancy and after delivery. This care is given to pregnant women to optimize quality health outcomes, such as normal birth weight, reduction in maternal and child death and low postpartum anemia [12]. More so, countries that have achieved success in improving maternal health care services and reducing maternal morbidity mortality overall, are still faced with the challenges of large inequities among various sections of the populations. The groups of women that are disadvantaged tend to have more morbidity and mortality, and inadequate access to safe motherhood services, acceptable and affordable health care services to enhance safe pregnancy and delivery [13]. Efforts have been made to reduce health inequities across all facets of the populations, on subnational, national and global levels, and ensure equal opportunities to all members of communities to achieve good health [14]. However, most health care systems are inequitable, benefiting the wealthy than the underprivileged [15].

There are significant disparities in maternal health care indicators across many geographical locations, maternal, economic, socio-demographic factors in many developing countries [16]. Whereas equity has been indicated as a prominent target within the health sectors, huge disparities exist in coverage of maternal and child health care services between the well-off and disadvantaged in low income countries. The inequalities and inequities across various strata of the society have become key determinants of maternal and child health [17, 18]. Obtaining equal access to maternal health care including antenatal, delivery and postnatal services, is one of the most critical concerns in public health programmes and policies shared in virtually everywhere in the world, and demands that women with the same maternal needs should receive the same access to health care services [19].

In this study, inequities in the determinants of major maternal health outcomes including antenatal care, institutional delivery with skilled birth attendance and utilization of modern contraception were examined using Benin Demographic and Health Survey (BDHS) dataset. We presented comparative analyses of the outcome variables in two separate BDHS to assess disparities in the utilization of these services.

## Methods

### Data extraction

Data for this study were derived from two rounds of Demographic and Health Survey in Benin that provided information on antenatal care, institutional delivery and contraceptive use. The datasets have one record for every eligible woman as defined by the household schedule. The questionnaire contains all the data collected from the individual woman for whom information on antenatal care, delivery and contraceptive usage and some variables from the household were elicited. The 2006 and 2012 Benin Demographic and Health Survey (BDHS) data contains 17,794 and 16,599 cases (units of analysis), which in this file is the woman. BDHS performed cross-sectional analyses using nationally representative data, to collect information on demographic, health, and nutrition indicators. The survey is majorly funded by the United States Agency for International Development (USAID). The two rounds of BDHS utilized a multi-stage, stratified sampling design, with households as the sampling unit. Within each sample household, all eligible women were interviewed [20].

### Outcome variables

In this study, we used four outcome measures of maternal health care utilization extracted from the BDHS. Firstly, we derived the; “number of antenatal care (ANC) visits during pregnancy”, this was grouped as 4 or more ANC visits vs below 4 ANC visits. ANC visits is a measure of skilled pregnancy care received by women during most recent pregnancy. Secondly, we extracted the “place of delivery (home vs health facility)”. This was measured as a binary outcome for 1, if a woman delivered in a health facility (where skilled delivery attention is available) and 0, if otherwise. In addition, postnatal care was measured by “respondents health’s checked after discharge/delivery at home” Lastly, women’s “contraceptive use”; was obtained as binary indicator taking 1 if the “woman ever used a contraceptive method” and 0, if otherwise.

### Explanatory variables

The utilization of ANC visits, facility-based delivery and contraceptive use are known to depend on a set of determinants, such as demographic, economic, other proximate and social factors. Empirical literature on the factors pertinent to maternal health care services basically helped to select the variables of study. These variables age of individual woman (15–19, 20–24, 25–29, 30–34, 35–39, 40–44 and 45–49 year), geographical region (Alibori, Atacora, Atlantique, Borgou, Collines, Couffo, Donga, Littoral, Mono, Queme, Plateau and Zou), type of residence (rural vs urban). Educational attainment was categorized as those having no formal education, primary, secondary and higher education. Religious beliefs included; Christianity, Islam, traditional and other religion, while access to health information was measured using frequency of reading newspaper or magazine, listening to radio and watching TV. The wealth scores is obtained by principal components analysis, based on a list of household assets as specified by DHS, which include, number of household members, wall and roof materials, floor types, access to potable water and sanitation, type of cooking fuel, ownership of television, radio, motorcycle, refrigerator amongst others. Based on the weighted wealth scores, households were grouped into five wealth quintiles; poorest, poorer, middle, richer and richest. Furthermore, parity was measured by the number of children ever born by each individual woman; categorized as 1–4 and > 4 children.

### Ethical considerations

We did the analyses using publicly available data from demographic health surveys. Ethical procedures were the responsibility of the institutions that commissioned, funded, or managed the surveys. All DHS surveys are approved by ICF international as well as an Institutional Review Board (IRB) in respective country to ensure that the protocols are in compliance with the U.S. Department of Health and Human Services regulations for the protection of human subjects.

### Statistical analysis

Summary statistics including percentage and means (±standard deviation) were used to examine the distribution of socio-demographic, economic distal and proximate maternal characteristics. To adjust for data representation, we used complex survey module (svyset) for all analyses to account for clustering, stratification and sample weight. In addition, the percentages of outcome variables were presented in bar chart. The factors associated with ANC visits, facility-based delivery, postnatal care and contraceptive use were examined using logistic regression models. The bivariate analysis conducted to examine the factors that were added in the multivariable regression models involved a simple regression with each explanatory variable. Therefore, factors, which were statistically significant in the crude regression models, were added in the multivariable regression models to adjust for possible confounders. An α level of 0.05 was considered statistically significant. All analyses were conducted using STATA 14.0.

## Results

### Sample characteristics

In this study, the characteristics of respondents were explored for 2006 and 2012 respectively. The mean ages of respondents were similar (29.0 ± 9.1/9.0) between the years of survey. The basic socio-demographic characteristics of the respondents were presented in Table 1.

**Table 1:** Characteristics of respondents. Benin DHS 2006–12

Variable	2006		2012	
	*n* (17,794)	%	*n* (16,599)	%
Age (Mean ± SD)	29.0 ± 9.1		29.0 ± 9.0	
15–19	3036	17.1	2922	17.6
20–24	3117	17.5	2820	17.0
25–29	3640	20.5	3147	19.0
30–34	2801	15.7	2720	16.4
35–39	2151	12.1	2185	13.2
40–44	1626	9.1	1667	10.0
45–49	1423	8.0	1138	6.9
Region				
Alibori	1197	6.7	1000	6.0
Atacora	1506	8.5	1476	8.9
Atlantique	1988	11.2	1866	11.2
Borgou	1535	8.6	1323	8.0
Collines	1234	6.9	1256	7.6
Couffo	1530	8.6	1225	7.4
Donga	893	5.0	950	5.7
Littoral	1831	10.3	1949	11.7
Mono	1196	6.7	1043	6.3
Quémé	2142	12.0	1811	10.9
Plateau	862	4.8	1046	6.3
Zou	1880	10.6	1654	10.0
Type of place of residence				
Urban	7471	42.0	7070	42.6
Rural	10,323	58.0	9529	57.4
Educational attainment				
No formal education	11,577	65.1	10,383	62.6
Primary	3460	19.4	2766	16.7
Secondary	2595	14.6	3219	19.4
Higher	162	0.9	231	1.4
Religion				
Christianity	9484	53.4	9226	55.6
Islam	3878	21.8	3919	23.6
Traditional	3178	17.9	2284	13.8
Others	1210	6.8	1170	7.0
Read newspaper/magazine				
Yes	1701	9.6	2140	12.9
No	15,957	90.4	14,459	87.1
Listen to radio				
Yes	14,499	81.7	10,525	63.4
No	3257	18.3	6074	36.6
Watch TV				
Yes	6398	36.1	7556	45.5
No	11,320	63.9	9043	54.5
Wealth index				
Poorest	3357	18.9	3139	18.9
Poorer	3347	18.8	3274	19.7
Middle	3448	19.4	3433	20.7
Richer	3753	21.1	3511	21.2
Richest	3889	21.9	3242	19.5
Parity				
1–4	8379	60.7	8377	66.9
> 4	5435	39.3	4145	33.1
Women decision making power				
Low	4140	35.1	2534	35.4
Moderate	7664	64.9	2741	38.2
High			1892	26.4
Currently working				
Yes	14,114	79.6	10,643	64.1
No	3628	20.4	5956	35.9
Sex of household head				
Male	14,353	80.7	13,326	80.1
Female	3441	19.3	3273	19.7

Prevalence of maternal health care utilization.

**Fig. 1:**
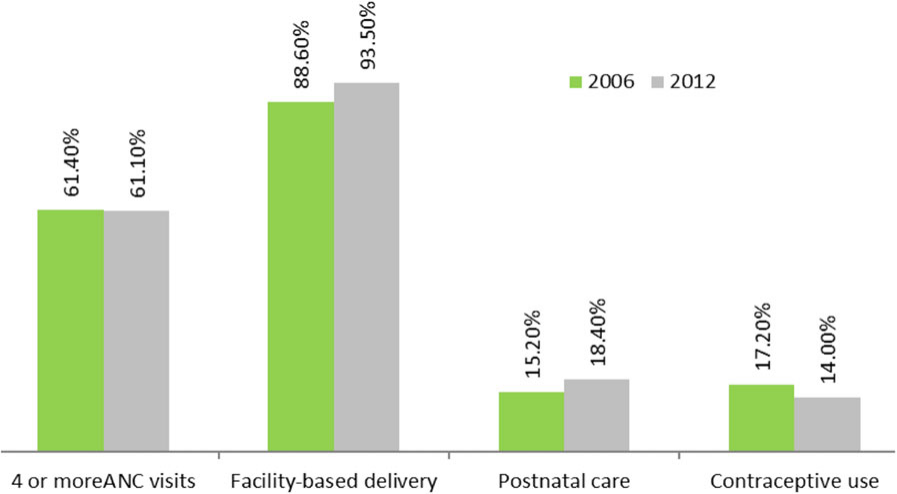
Prevalence of antenatal care, contraceptive use and facility-based delivery among women of reproductive age in Benin

In this study, four outcomes were measured namely; antenatal care of at least 4 visits, facility-based delivery, postnatal care and utilization of contraceptive methods. The percentage of 4 or more antenatal visits was 61.4% in 2006, and 61.1% in 2012, which showed that there was no increase in the level of antenatal care visits over time. Facility-based delivery was reported as 88.6% in 2006 which had 4.9% increase by 2012. Postnatal care 15.2% in 2006, but increased to 18.4% in 2012. Further, the percentage of contraceptive use was 17.2% in 2006, however reduced to 14% in 2016 (see Fig. 1 for details).

The results showed that women aged 35–49 years had increase in the odds of facility-based delivery, compared to women aged 15–19 years. Further, women aged 45–49 years had 45% significant reduction in the odds of contraceptive use, compared to women aged 15–19 years after adjusting for other covariates (OR = 0.55; 95% CI = 0.33–0.93). Also, geographical region was significantly associated with ANC visits, facility-based, postnatal care and contraceptive use. Rural women had 17% reduction in the odds of contraceptive use, compared to the urban women after adjusting for other covariates (OR = 0.83; 95% CI = 0.70–0.98). Educated women were more likely to utilize ANC visits, facility-based and postnatal care, compared to women with no formal education after adjusting for other covariates.

Women with higher wealth index had significant increase in the odds of adequate ANC visits, facility-based delivery and contraceptive use. Number of children ever born was significantly associated with maternal health care services; women who had above 4 children had significant reduction in adequate ANC visits and facility-based delivery respectively, compared to women who had 1–4 children (ANC- OR = 0.71; 95% CI = 0.61–0.82; facility-based delivery- OR = 0.59; 95% CI = 0.48–0.72). Notwithstanding, women who had above 4 children were 1.48 times as likely to have contraceptive use, compared to women who had 1–4 children (OR = 1.48; 95% CI = 1.27–1.71). Women with moderate decision-making power were 1.26 times as likely to have contraceptive use, compared to women with low decision-making power. Also, women from female headed households were 1.58 times more likely to utilize facility-based delivery, compared to women from male headed households (OR = 1.58; 95% CI = 1.18–2.11). See Table 2 for details.

**Table 2:** Odds ratios antenatal care visits, skilled birth delivery and contraceptive use for the years 2006 and 2012

Variable	ANC		Facility-based delivery		Postnatal care		Contraceptive use	
	2006	2012	2006	2012	2006	2012	2006	2012
Age								
15–19	1.00	1.00	1.00	1.00	1.00	1.00	1.00	1.00
20–24	0.95 (0.69–1.29)	1.19 (0.93–1.52)	0.95 (0.62–1.47)	0.83 (0.31–2.22)	0.90 (0.61–1.33)	0.89 (0.54–1.48)	1.10 (0.69–1.75)	1.04 (0.85–1.28)
25–29	0.92 (0.68–1.26)	1.25 (0.98–1.58)	1.25 (0.83–1.88)	0.84 (0.32–2.21)	0.83 (0.57–1.21)	1.12 (0.70–1.79)	1.06 (0.67–1.68)	1.19 (0.97–1.47)
30–34	1.07 (0.77–1.49)	1.08 (0.85–1.37)	1.49 (0.96–2.32)	0.67 (0.23–1.91)	0.87 (0.58–1.29)	1.09 (0.67–1.77)	1.10 (0.68–1.77)	1.04 (0.83–1.29)
35–39	1.20 (0.85–1.69)	1.05 (0.82–1.35)	1.81 (1.10–2.97)	1.21 (0.42–3.50)	1.04 (0.67–1.59)	1.25 (0.76–2.04)	1.10 (0.67–1.80)	1.33 (1.06–1.68)
40–44	1.12 (0.76–1.66)	1.08 (0.81–1.43)	3.45 (1.96–6.10)	1.41 (0.48–4.18)	0.70 (0.43–1.15)	1.14 (0.64–2.01)	0.92 (0.56–1.52)	1.31 (1.04–1.65)
45–49	1.07 (0.72–1.59)	1.29 (0.86–1.94)	8.39 (4.74–14.83)	14.5 (3.55–59.41)	0.91 (0.52–1.61)	0.59 (0.25–1.37)	0.55 (0.33–0.93)	0.90 (0.69–1.16)
Region								
Alibori	1.00	1.00	1.00	1.00	1.00	1.00	1.00	1.00
Atacora	1.56 (0.99–2.46)	1.09 (0.73–1.60)	1.68 (0.99–2.82)	1.07 (0.49–2.33)	1.29 (0.70–2.38)	2.72 (1.59–4.66)	1.98 (1.16–3.36)	1.53 (0.79–2.96)
Atlantique	3.14 (2.01–4.92)	0.87 (1.29–2.71)	31.1 (15.64–61.85)	5.37 (2.52–11.41)	0.22 (0.10–0.45)	1.43 (0.89–2.32)	5.18 (3.03–8.86)	0.81 (0.48–1.37)
Borgou	1.69 (1.02–2.80)	0.93 (0.65–1.34)	1.14 (0.65–2.00)	0.97 (0.54–1.72)	0.37 (0.20–0.71)	2.85 (1.71–4.74)	1.63 (0.95–2.81)	1.61 (1.02–2.55)
Collines	2.02 (1.28–3.19)	1.30 (0.87–1.94)	5.63 (2.96–10.69)	3.39 (1.38–8.36)	0.91 (0.47–1.75)	3.77 (2.82–6.24)	5.12 (3.04–8.64)	1.67 (0.96–2.92)
Couffo	1.55 (0.98–2.43)	2.07 (1.39–3.11)	2.64 (1.51–4.63)	1.78 (0.76–4.19)	1.04 (0.52–2.10)	1.29 (0.72–2.32)	1.67 (0.94–2.96)	1.58 (0.95–2.62)
Donga	1.57 (0.97–2.55)	0.92 (0.61–1.39)	1.85 (1.01–3.82)	1.46 (0.61–3.53)	1.80 (1.01–3.21)	1.60 (0.86–3.00)	1.04 (0.59–1.84)	0.47 (0.26–0.84)
Littoral	3.88 (2.24–6.73)	1.49 (0.98–2.26)	7.80 (3.13–19.14)	1.50 (0.46–4.89)	1.29 (0.53–3.13)	2.27 (1.36–3.80)	2.69 (1.57–4.62)	1.32 (0.81–2.17)
Mono	2.32 (1.44–3.72)	2.74 (1.77–4.24)	9.24 (4.46–19.14)	14.75 (4.74–45.88)	1.23 (0.63–2.42)	2.47 (1.41–4.32)	1.55 (0.89–2.72)	0.39 (0.22–0.67)
Quémé	2.60 (1.69–3.99)	2.84 (1.94–4.18)	28.60 (14.52–56.33)	35.33 (4.67–267.4)	0.41 (0.20–0.85)	2.30 (1.46–3.63)	3.00 (1.81–4.96)	0.62 (0.38–0.99)
Plateau	2.07 (1.31–3.29)	0.74 (0.49–1.13)	4.38 (2.46–7.81)	1.38 (0.61–3.12)	0.77 (0.35–1.70)	1.83 (0.76–4.40)	0.98 (0.53–1.81)	1.33 (0.79–2.24)
Zou	2.85 (1.81–4.49)	2.42 (1.64–3.55)	19.49 (10.48–36.24)	8.47 (3.66–19.61)	0.57 (0.28–1.17)	1.03 (0.61–1.73)	1.96 (1.15–3.32)	1.32 (0.78–2.23)
Type of place of residence								
Urban	1.00	1.00	1.00	1.00	1.00	1.00	1.00	1.00
Rural	1.02 (0.86–1.24)	1.01 (0.85–1.19)	0.93 (0.64–1.34)	0.68 (0.42–1.10)	0.82 (0.61–1.10)	0.86 (0.67–1.09)	0.83 (0.70–0.98)	1.17 (0.94–1.47)
Educational attainment								
No formal education	1.00	1.00	1.00	1.00	1.00	1.00	1.00	1.00
Primary	1.29 (1.08–1.53)	1.63 (1.39–1.92)	2.12 (1.56–2.89)	1.97 (1.20–3.26)	1.04 (0.75–1.45)	0.91 (0.68–1.23)	1.42 (1.22–1.65)	1.33 (1.14–1.56)
Secondary	1.28 (0.91–1.79)	2.13 (1.69–2.68)	5.79 (1.89–17.72)	3.40 (1.25–9.24)	1.08 (0.57–2.08)	1.12 (0.75–1.68)	1.95 (1.59–2.38)	1.87 (1.55–2.25)
Higher		2.60 (0.90–7.54)			15.35 (0.95–247.2)	1.16 (0.24–5.55)	1.16 (0.68–1.97)	2.09 (1.35–3.26)
Religion								
Christianity	1.00	1.00	1.00	1.00	1.00	1.00	1.00	1.00
Islam	0.76 (0.61–0.95)	0.80 (0.67–0.96)	0.60 (0.45–0.81)	0.59 (0.39–0.90)	0.84 (0.55–1.29)	0.87 (0.68–1.12)	0.83 (0.68–1.02)	1.29 (1.08–1.55)
Traditional	0.81 (0.67–0.97)	0.68 (0.57–0.82)	0.48 (0.35–0.64)	0.57 (0.32–1.02)	1.30 (0.97–1.75)	1.07 (0.77–1.49)	0.86 (0.69–1.08)	0.80 (0.63–0.99)
Others	0.80 (0.64–0.98)	0.70 (0.56–0.88)	0.53 (0.39–0.73)	0.56 (0.34–0.92)	1.33 (0.91–1.94)	0.84 (0.58–1.3)	0.71 (0.55–0.92)	0.64 (0.46–0.88)
Read newspaper/magazine	1.19 (0.78–1.82)	0.89 (0.68–1.16)	0.85 (0.37–1.94)	0.62 (0.22–1.76)	1.07 (0.53–2.18)	1.31 (0.83–2.05)	1.46 (1.17–1.81)	0.89 (0.75–1.07)
Listen to radio	1.36 (1.17–1.57)	0.96 (0.84–1.10)	1.38 (1.13–1.70)	1.29 (0.93–1.78)	0.98 (0.76–1.26)	1.06 (0.83–1.36)	1.39 (1.14–1.68)	1.16 (1.01–1.34)
Watch TV	1.39 (1.18–1.63)	1.31 (1.13–1.52)	1.49 (1.13–1.97)	1.69 (1.20–2.37)	1.56 (1.13–2.15)	0.99 (0.79–1.23)	1.28 (1.08–1.52)	1.38 (1.16–1.65)
Wealth index								
Poorest	1.00	1.00	1.00	1.00	1.00	1.00	1.00	1.00
Poorer	1.39 (1.18–1.63)	1.36 (1.15–1.60)	2.02 (1.67–2.45)	1.55 (1.13–2.13)	1.22 (0.95–1.56)	0.82 (0.61–1.10)	1.27 (1.01–1.60)	1.06 (0.83–1.34)
Middle	1.86 (1.58–2.20)	1.78 (1.51–2.11)	2.97 (2.38–3.72)	2.36 (1.63–3.43)	0.95 (0.72–1.25)	0.91 (0.67–1.22)	1.40 (1.11–1.76)	1.19 (0.94–1.51)
Richer	2.64 (2.19–3.17)	2.62 (2.15–3.20)	7.36 (5.31–10.18)	7.14 (4.08–12.49)	1.06 (0.76–1.48)	0.86 (0.61–1.23)	1.48 (1.16–1.87)	1.10 (0.85–1.43)
Richest	5.19 (3.96–6.80)	3.71 (2.78–4.94)	13.04 (7.10–23.95)	21.63 (7.30–64.08)	1.67 (1.09–1.60)	1.16 (0.79–1.70)	2.07 (1.59–2.71)	1.14 (0.85–1.54)
Parity								
1–4	1.00	1.00	1.00	1.00	1.00	1.00	1.00	1.00
> 4	0.71 (0.61–0.82)	0.93 (0.84–1.04)	0.59 (0.48–0.72)	0.78 (0.58–1.04)	1.01 (0.84–1.21)	1.03 (0.84–1.25)	1.48 (1.27–1.71)	1.06 (0.94–1.21)
Women decision making power								
Low	1.00	1.00	1.00	1.00	1.00	1.00	1.00	1.00
Moderate	1.04 (0.91–1.19)	1.02 (0.86–1.21)	0.91 (0.74–1.12)	0.96 (0.70–1.31)	0.97 (0.74–1.27)	0.76 (0.55–1.06)	1.26 (1.10–1.44)	0.97 (0.81–1.18)
High		1.22 (1.01–1.47)		1.47 (1.03–2.10)		1.12 (0.77–1.63)		1.04 (0.85–1.27)
Currently working	1.10 (0.94–1.28)	1.71 (1.49–1.95)	0.69 (0.55–0.86)	2.52 (1.51–4.22)	1.09 (0.78–1.53)	0.87 (0.71–1.07)	0.97 (0.87–1.08)	1.37 (1.21–1.56)
Sex of household head								
Male	1.00	1.00	1.00	1.00	1.00	1.00	1.00	1.00
Female	1.11 (0.92–1.33)	1.09 (0.92–1.30)	1.58 (1.18–2.11)	2.04 (1.14–3.67)	1.11 (0.82–1.51)	1.02 (0.75–1.37)	1.13 (1.00–1.26)	1.12 (0.98–1.28)

*N.B.* Numbers represent odds ratios. Ref = reference category

For 2012, women aged 45–49 years had higher odds of facility-based, compared to women aged 15–19 years after adjusting for other covariates. Respondents aged 35–44 years also had increase in the odds of contraceptive use, compared to those aged 15–19 years. The region and religion of respondents was significantly associated with adequate ANC visits, facility-based delivery, postnatal care and contraceptive use. The respondents with high decision-making power were 1.47 times as likely to have facility-based delivery, compared to women with low decision-making power after adjusting for other covariates (OR = 1.47; 95% CI = 1.03–2.10). Further, women who were currently working or employed had higher odds of ANC visits, facility-based delivery and contraceptive use. Women from female headed households were 2.04 times as likely to have facility-based delivery, compared to other counterpart after adjusting for other covariates (OR = 2.04; 95% CI = 1.14–3.67).

## Discussion

### Main findings

This study has become the foremost to explore and examine prominent indicators of maternal health care service utilization in Benin, and thus utilized two rounds of nationally representative data set from 2006 and 2012 surveys. The main outcome measures under study were ANC visits, institutional delivery, postnatal care and contraceptive use among women of reproductive age. The prevalence of ANC at least 4 or more visits, facility-based delivery, postnatal care and contraceptive use among women of reproductive age were relatively the same in both rounds of survey. The estimates are consistent with literature from other sub-Saharan Africa countries where maternal health care indicators need more improvement [21–23]. Furthermore, socio-demographic, economic and proximate determinants were the main predictors of maternal healthcare services. Age, geographical region, place of residence, level of education, religious beliefs, use of media, wealth index and parity were significant predictors for the disparity in access to skilled pregnancy care and fertility control [22–25].

In addition, this study found disparities in the demographic, social, economic and proximate factors associated with the utilization of maternal health care services. The SDGs are known to support reduction in inequalities and ensure health for all populations. In the light of the above, beyond the utilization of maternal health care, also to reach the most disadvantaged group of the women, vis-a-vis the use of maternal care services must be considered to achieve the set goals [26]. The findings in this study showed that women with lower educational level, inaccessibility of the media (newspaper, radio and television), low economic class, rural dwellers amongst others are less likely to utilize maternal healthcare services. This revealed disproportionate share of maternal healthcare services by certain class of people despite of the high demand. The results were in line with previous studies in other countries [27–30].

Though there are interventions to improve coverage of maternal and child health care services in sub-Saharan Africa, the disparity in access and use by several factors such as place of residence, use of media, geographical region, educational attainment, age of women and wealth quintile amongst others has remained persistently high in the two rounds of data utilized [30, 31]. Explaining differentials in accessing maternal and reproductive care services is a critical issue, because several other contributory factors, including distance, cultural beliefs or practices, health care seeking behavior, affordability and need for services must also be considered [31]. The findings of this study were consistent with previous studies that reported that improvement in economic status was connected to better use of maternal health services [10]. Again, religious beliefs and residency usually indicate cultural background and influence norms, values and beliefs in relation to women’s status, service use and childbirth. Previous studies have reported low levels of maternal healthcare utilization among ethnic minority women in rural areas [32–34]. Also, maternal decision-making power was associated with health care behaviors. Women’s empowerment enhance the knowledge and need of health care and awareness of services which can improve through behavior change communication and increase the ability of a woman to have positive health care-seeking behavior. Overall, our study is consistent with the findings from a review of numerous studies in developing countries that women’s empowerment is positively associated with the use of maternal health care services [35].

### Strengths and limitations

The main strength of this study is that it leverages on nationally representative data collected through a consistent methodology in 2006 and 2011–12. In addition, this is the foremost nationwide analyses that explores antenatal visits, facility-based delivery, postnatal and contraceptive utilization in Benin, and as such could serve as benchmark and stimulus for further nationwide studies on related subjects. Nonetheless, cross sectional data are unable to sufficiently establish causality, again due to self-report method of eliciting information from respondents, there could be possibility of recall bias which could affect the level of utilization of maternal health care services reported in this study.

## Conclusion

This study has identified the importance of vital maternal care services and associated socio-demographic, economic and proximate factors disparities against the backdrop of poor maternal health indicators in Benin. The findings showed consistent differentials in the use of key maternal health services, such as ANC visits, facility-based delivery, postnatal care and contraceptive use, in favor of women in urban, educated, high economic status and use of media. Though the study revealed disparities in selected determinants of maternal care services in Benin, however, there are other factors such as environmental conditions, governance, culture, infrastructure and availability of medical equipment and personnel that play vital role in reduction of these differences. Hence, the findings would guide stakeholders in health care system to address the unequal access in health care services. As we strive for universal health coverage, health care interventions, programmes and policies should be strengthened to enhance maternal care services utilization, and tackle the disparities in the utilization of maternal care services, especially for the disadvantaged who live in hard-to-reach rural areas in Benin. The Benin government needs to create strategies that cover both the supply and demand side interventions, specifically to reach the uneducated, living in remote areas with inadequate resources to have access to health care services. Such strategy must go beyond any specific intervention for maternal health care to accommodate a wider developmental agenda and human capital development.

## Abbreviations

ANC: Antenatal care
DHS: Demographic Health Survey
IRB: Institutional Review Board
MMR: Maternal Mortality Ratio
SDGs: Sustainable Development Goals
SSA: Sub-Saharan Africa
TBA: Traditional Birth Attendant
USAID: United States Agency for International Development
WHO: World Health Organization
